# Designing Imidazolium-Mediated Polymer Electrolytes for Lithium-Ion Batteries Using Machine-Learning Approaches: An Insight into Ionene Materials

**DOI:** 10.3390/polym17152148

**Published:** 2025-08-06

**Authors:** Ghazal Piroozi, Irshad Kammakakam

**Affiliations:** Department of Chemistry, School of Sciences and Humanities, Nazarbayev University, Astana 010000, Kazakhstan; ghazal.piroozi@nu.edu.kz

**Keywords:** poly(ionic liquids), polymer electrolytes, lithium-ion batteries, machine learning, CatBoost model

## Abstract

Over the past few decades, lithium-ion batteries (LIBs) have gained significant attention due to their inherent potential for environmental sustainability and unparalleled energy storage efficiency. Meanwhile, polymer electrolytes have gained popularity in several fields due to their ability to adapt to various battery geometries, enhanced safety features, greater thermal stability, and effectiveness in reducing dendrite growth on the anode. However, their relatively low ionic conductivity compared to liquid electrolytes has limited their application in high-performance devices. This limitation has led to recent studies revolving around the development of poly(ionic liquids) (PILs), particularly imidazolium-mediated polymer backbones as novel electrolyte materials, which can increase the conductivity with fine-tuning structural benefits, while maintaining the advantages of both solid and gel electrolytes. In this study, a curated dataset of 120 data points representing eight different polymers was used to predict ionic conductivity in imidazolium-based PILs as well as the emerging ionene substructures. For this purpose, four ML models: CatBoost, Random Forest, XGBoost, and LightGBM were employed by incorporating chemical structure and temperature as the models’ inputs. The best-performing model was further employed to estimate the conductivity of novel ionenes, offering insights into the potential of advanced polymer architectures for next-generation LIB electrolytes. This approach provides a cost-effective and intelligent pathway to accelerate the design of high-performance electrolyte materials.

## 1. Introduction

**Figure 1:**
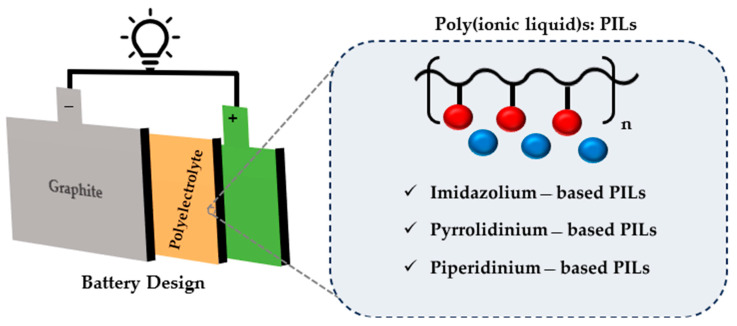
Schematic of a lithium-ion battery illustrating the cathode, anode, electrolyte, and common types of poly(ionic liquid) electrolytes.

As the world moves towards green energy, storage systems, including hydrogen storage [1], pumped hydrogen storage [2], flow batteries [3], and especially lithium-ion batteries [4,5] are increasingly in demand. A revolution in electronic devices began 30 years ago when Sony successfully commercialized the world’s first lithium-ion battery (LIB) [4]. Since then, LIBs have gained significant attention due to their inherent potential for environmental sustainability and unparalleled energy storage efficiency. LIBs application is not limited to portable electronics; they are also used in various energy sectors and devices, including but not limited to hybrid and big electric vehicles, remote-controlled devices, solar energy equipment, medical tools, and more. Their use is also increasing in the aerospace and military industries [4,6,7]. LIBs are a part of the rechargeable family of batteries, similar to other batteries, and consist of four main components: the anode, cathode, electrolyte, and separator (Figure 1).

To enhance lithium-ion battery performance, electrolytes have garnered significant attention as a key component of batteries. Organic electrolytes consisting of linear and alkyl carbonates are well-known and utilized for their wide operating voltage. Meanwhile, polymer electrolytes have gained popularity in the fields of electrical, aerospace, automotive, and electronics due to their ability to adapt to various battery geometries, improved safety features, low manufacturing costs, higher thermal stability, and effectiveness in reducing dendrite growth on the anode. Polymer electrolytes are composed of polymer matrices and lithium salts that were initially introduced during the 1970s [3,4]. Despite these advantages, polymer electrolytes are highly volatile and flammable, posing significant safety risks. Consequently, research on non-flammable electrolytes with a high lithium-ion transfer number is ongoing to enhance the safety and efficiency of lithium batteries [6]. Solutions being investigated include solid ceramic electrolytes, polymer electrolytes (solid, gel, and composite), aqueous lithium-ion batteries, fluorinated structures, and ionic liquids [2,7,8].

Lithium batteries rely on liquid electrolytes, which have the advantages of high ionic conductivity and superior wetting performance at the electrode surface [9]. Ionic liquids (ILs) are considered emerging potential candidates for replacing carbonate-based electrolytes in the market. ILs are salts with a melting point below 100 °C that have high chemical and thermal stability, as well as very low or zero vapor pressure. Such characteristics enable room-temperature ILs to be ideal options for a broad range of uses, particularly in electrochemical devices like LIBs [9,10]. In general, ILs are a class of molten salts consisting of an array of asymmetric organic cations and organic or inorganic anions. The most widely studied ILs are ammonium-based ILs such as imidazolium, pyrrolidinium, and quaternary ammonium-based ILs [11]. At the same time, polymerized ionic liquids (PILs) are a subclass of polyelectrolytes formed by polymerizing ionic liquid monomers, where either the cation, anion, or both are covalently bound to the polymer backbone. These materials combine the desirable properties of traditional free ionic liquids, such as high ionic conductivity and electrochemical stability, with the mechanical robustness, processability, and safety advantages of polymeric systems. Among various structural types, imidazolium-based PILs are the most widely studied due to their favorable electrochemical properties, thermal stability, and ease of synthesis [12].

On the other hand, the emergence of machine learning (ML) has entered the material science field into a new era. ML has accelerated the process of material discovery, design, and optimization by employing large datasets and advanced algorithms [13]. Most interestingly, ML models can make precise predictions by identifying patterns within the existing datasets while avoiding the pressing challenges of experimentation, including related timeframe and costs. Although ML models are simple mathematical calculations, the complex nature of materials, particularly polymers, presents intricate challenges in developing ML models. Therefore, recent advances in ML models have been widely examined, with more contemporary applications in macromolecular studies, discussing group contributions and chemical structures via SMILES representation. Furthermore, recent ML advancements in the polymer field include accelerated polymer simulations [14], prediction of polymer properties [12,15], adhesion strength prediction [16], and polymer discovery and design [17,18].

Technically, large datasets are necessary to train machine learning (ML) models. Nevertheless, a recent study employed a self-supervised strategy using a graph neural network (GNN) to predict polymer properties solely based on polymer structure data, as reported by Gao et al. [19]. Those results paved the way for tuning to be possible on smaller datasets for specific property prediction tasks, thanks to the pre-trained GNN. In data-scarce scenarios, the results further indicated that the ensemble pre-training approach outperforms other approaches, for electron affinity and ionization potential root mean square error (RMSE) was reduced by 28.39% and 19.09%, respectively. Kazemi-Khasragh et al. [20] employed a transfer strategy approach to focus on the prediction of mechanical and thermal properties of linear polymers. Firstly, the artificial neural network (ANN) algorithm was pre-trained to predict heat capacity at constant pressure (Cp) using 124 data points, then the pre-trained model was fine-tuned to predict specific capacity, shear modulus, flexural stress strength, and dynamic viscosity. In addition to transfer learning, researchers in this field have also employed other machine learning models. Babbar et al. [21] developed three ML models, namely ANN, convolutional neural network (CNN), and ridge regression (RR), to predict the glass transition temperature (Tg) of polymers. In this study, two types of molecular fingerprints were used as input features: physicochemical and topological fingerprints. The former was extracted from RDKit and used as input for RR and ANN models, while the latter was derived from the SMILES representation using one-hot encoding and used as input for the CNN model. The results highlighted the reasonable performance of RR compared to powerful non-linear models of ANN and CNN. Ascencio-Medina et al. [22], by analyzing a dataset of 86 polymers, investigated the dielectric permittivity in polymers. They employed a genetic algorithm to select the most relevant descriptors from a set of 1273 descriptors. Then, by using a gradient boosting regressor (GBR), the dielectric constant was predicted. This model achieved high accuracy with a correlation coefficient (R^2^) of 0.938 and 0.822 for the training and test sets, respectively.

Electrical conductivity is another essential property of polymers, which plays a vital role in their ability to transport charge. However, limited research has been conducted in this field for predicting the conductivity of polymers. Hatakeyama-Sato et al. [23] constructed a 104-entry database of lithium-conducting solid polymers, the largest of its kind. The authors employed a transfer-learned graph neural network (GNN) for predicting the conductivity of electrolytes, resulting in a mean absolute error (MAE) of less than 1. The unbiased predictions of the model led to the discovery of superionic conductors with ionic conductivities of about 10^−3^ at room temperature. Li et al. [24], incorporated GNNs with quantum calculations to develop an automatic system for identifying potential ionic liquids (ILs) for ionic liquid polymer electrolytes (IPEs). Firstly, based on the ensemble learning of support vector machine (SVM), random forest (RF), XGBoost, and graph convolutional neural networks (GCNN), the phase of ILs was predicted. After identifying the IL candidates, the datapoints were classified based on conductivity type (σ ≥ 5 and σ < 5). XGBoost and SVM performed better than other models. According to the results, the median values reported for the groups with σ < 5 and σ ≥ 5 are 1.8 and 9.1 mS cm^−1^, respectively. Most recently, Bradford et al. [25] constructed a chemistry-informed ML workflow that predicted the conductivity of solid polymers by using chemical structure, temperature, molecular weight (Mw), and salt concentration. The results were primarily developed using the novel approach of ChemArr, which was benchmarked against two other machine learning models, Chemprop and XGBoost. Among the developed models, XGBoost exhibited weak performance, while ChemArr outperformed other models by showing low MAE and a high Spearman rank correlation coefficient.

Taking into account the importance of designing polymerized ionic liquids (PILs) as polyelectrolytes in LIB applications, in this study, we have exclusively considered the structural and electrical performance of imidazolium-based poly (IL)s via machine learning (ML) approaches. As such, four impressive ML models, namely: CatBoost, RF, XGBoost, and LightGBM, have been meticulously selected to predict ionic conductivity of imidazolium-based PILs both in the form of solid polymer electrolytes (SPE) as well as gel polymer electrolytes (GPE).

Our study focuses on predicting ionic conductivity, which is a complex property influenced by multiple interdependent physical factors such as temperature, polymer structure (e.g., chain flexibility, polarity, and molecular weight), and ion transport pathways. These relationships are typically non-linear and high-dimensional.

The models we selected are namely: CatBoost, RF, XGBoost, and LightGBM, which are all ensemble tree-based algorithms known for:

- Handling non-linear interactions, which are common in conductivity–structure–temperature relationships;
- Robustness to multicollinearity, which is useful given the correlated chemical descriptors (as shown in the heatmap in Figure 6);
- Compatibility with small-to-medium datasets, which is important in polymer science, where experimental data is often limited;
- Built-in feature importance interpretation, helping us identify which physical factors (e.g., temperature, refractivity descriptors, surface area contributions) most strongly influence ionic conductivity.

Specifically, CatBoost was chosen as our primary model because it handles categorical and numerical features effectively, and minimizes overfitting through ordered boosting and permutation-based training features that benefit the relatively small and noisy nature of polymer datasets. Furthermore, prior literature [24,25,26,27,28] also demonstrates that such gradient boosting models are highly competitive for prediction tasks, especially when domain physical descriptors are included as model inputs. In summary, our selected ML models are well-aligned with the non-linear, multi-variable nature of conductivity prediction in imidazolium-functionalized ionene polymers, and their performance and interpretability make them particularly appropriate for advancing screening in this context.

**Figure 2:**
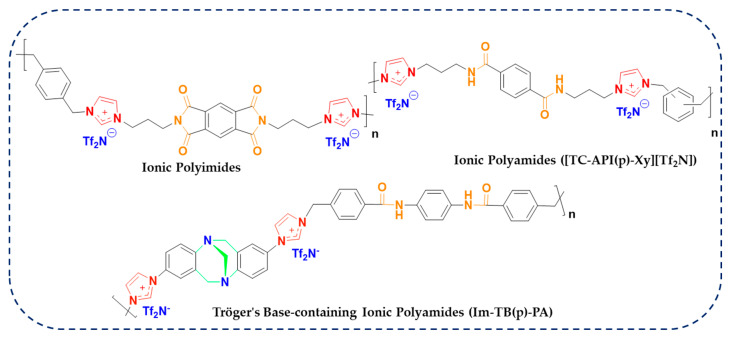
Chemical structures of novel ionene materials having polymeric backbones of amide and imide functionalities used for the ML approach, quantifying the conductivity data in this study.

**Figure 3:**
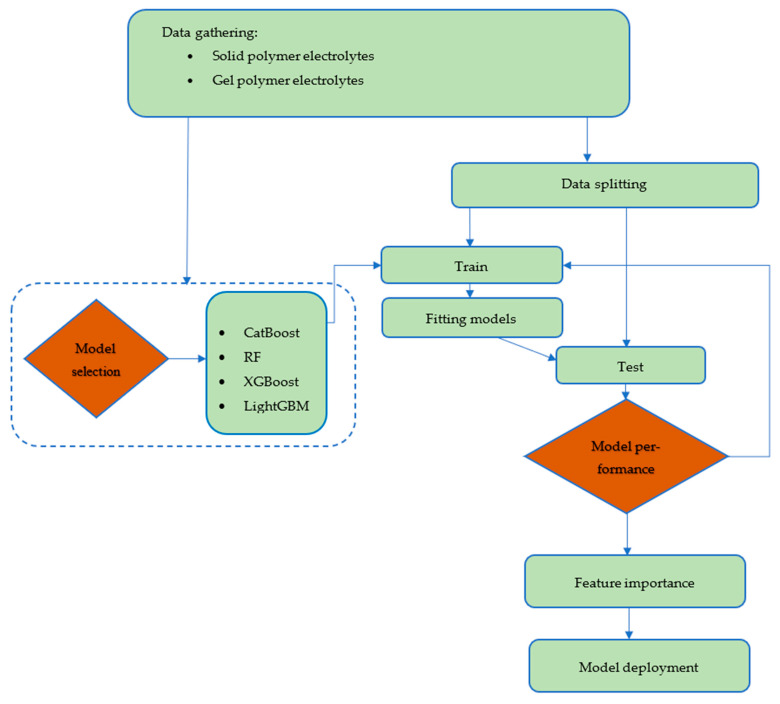
Flowchart of the ML model development process in this study.

Regarding this purpose, input features, including chemical structure and temperature, were gathered from the literature. The models were trained and tested on the dataset that was collected. Afterwards, the importance of the input features on conductivity prediction was investigated by the best-performing model. After training and testing the models, the best-performing model was used to predict conductivity for a new set of data points. Most importantly, we have aimed to anticipate the potential candidacy of ionene materials having conventional polymeric functional groups such as amides and imides. Specifically, we have focused on investigating the electrochemical performance in terms of conductivity data of our recently developed new type of ionic polymer, in which imidazolium cations are tethered within the rigid polyimide (PI) and polyamide (PA) substructures, as depicted in Figure 2 [29,30,31]. Overall, this study is organized into four sections. First, an introduction to lithium batteries, polymer electrolytes, and ML studies is provided. Then, in the methodology section, a description of each of the developed models is given. Moreover, this section describes the data gathering process. In the Results and Discussion section, the performance of the models will be assessed using various graphical and statistical methods. Additionally, a section is dedicated to the employment of ML models in predicting conductivity for ionenes with robust polymeric backbones. Ultimately, conclusions are drawn toward the insight of the usage of ionene polymeric materials as polymer electrolytes for LIB applications. The flowchart of the employed strategies is depicted in Figure 3.

## 2. Materials and Methods

### 2.1. Model Developments

Four ML models of CatBoost, RF, XGBoost, and LightGBM were employed in this study. These models will be briefly discussed in the sections below.

#### 2.1.1. CatBoost

**Figure 4:**
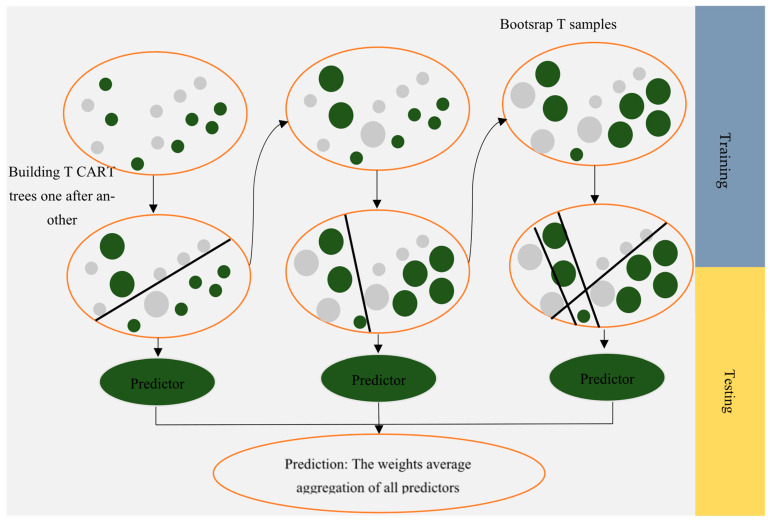
Schematic of the CatBoost algorithm.

CatBoost, as an open-source gradient boosted decision tree (GBDT) method, can handle categorical features properly. The main difference between the GBDT model and CatBoost is that, instead of preprocessing time, CatBoost deals with categorical features during training time. Prokhorenkova et al. [32] introduced target statistics (TS) as an efficient strategy for handling categorical features while losing minimum information. In particular, CatBoost permutes the dataset for each example and calculates an average label value based on the category value placed previously in the permutation. Moreover, CatBoost is different from GBDT in terms of feature combinations. Almost all categorical features should be combined to make a new one. CatBoost considers combinations greedily when building a new split for the tree. For the second and following splits, CatBoost combines all combinations (known as “combinations preset”) with all remaining categorical features. Every split in the tree is considered a category with two values, and they are added together in combination. Additionally, compared to GBDT, CatBoost performs an unbiased boosting with categorical features. To convert categorical features into numerical values with the TS method, the distribution will vary from the original one. In traditional GBDT methods, the deviation of this distribution will result in a deviation in the solution, which is an inevitable problem for GBDT. A random permutation of the training data is generated in CatBoost. To improve the robustness of the algorithm, multiple permutations will be used by sampling a random permutation and obtaining its slope. Calculating statistics based on permutations is similar to those calculated for classification features. Different permutations are used to train distinct models, and hence, using multiple permutations will not lead to overfitting [33]. Figure 4 shows a schematic of the CatBoost model.

#### 2.1.2. Random Forest

Breiman first developed RF [34]. The initial goal of RF algorithm development was to solve unsupervised regression and classification problems. This technique involves building multiple independent decision trees, also referred to as ensemble trees, training them based on the desired dataset, and then predicting the target parameter. In this algorithm, bootstrap resampling is used to prevent overfitting, a resampling method that relies on replacement. A bootstrap set is created by replacing several samples with repeated samples from the initial data. The RF algorithm then builds each tree using a bootstrap set. Therefore, since the trees were constructed on varied datasets, their predictions would be different. The next step is to aggregate all the trees, and the final prediction is obtained by averaging each tree’s predictions. With the RF model, the degree of importance of each feature and the proximity of samples in pairs can be determined [35,36].

#### 2.1.3. XGBoost

XGBoost is a popular boosting tree algorithm based on a decision tree, also known as a classification and regression tree (CART) [37]. CART divides the dataset into two subsets at each level according to the boundary of a variable for regression tasks, until it reaches the maximum tree depth specified by users. Searching for the best solutions is done by an algorithm for a range of variables to minimize the cost function. Then the prediction is the average of the target value of all samples in a subset. CART trees can be prone to overfitting without proper regularization. One strategy for this is called ensemble packing of a group of estimators, in other words, multiple CART models. XGBoost continues to add and train new trees to accommodate the remaining errors from the last iteration. Then, a predicted value is assigned to each sample by summing all the scores of the corresponding leaves. The advantage of XGBoost in performance is its reliable objective function for tree creation [38,39].

#### 2.1.4. LightGBM

LightGBM is a new GBDT algorithm that was first released by Microsoft by Ke et al. [40] in 2017, which has been employed in a variety of data mining tasks such as regression, classification, and sorting. The LightGBM algorithm incorporates several novel techniques, including gradient-based one-side sampling (GOSS), exclusive feature bundling (EFB), and a depth-constrained histogram and leaf-based growth strategy. Light GBM grows the tree vertically, while other algorithms, such as XGBoost and GBDT, grow trees horizontally. The mechanism of GOSS involves retaining all large gradient samples while performing random sampling on small gradient samples, based on their proportion. The main idea of EFB is to divide the features into a smaller number of unique mutual bundles.

#### 2.1.5. Evaluation Metrics

The performance of the conductivity prediction models was evaluated by the following metrics: mean absolute error (MAE), root mean square error (RMSE), and determination coefficient (R^2^). Commonly, these metrics are used in regression tasks; the higher the R^2^ value and the lower the MAE and RMSE value, and the better the accuracy of the models.

(1) $$\mathrm{RMSE}=\sqrt{\frac{1}{2n}\sum_{i=1}^{n}{\left(y_{i}-{\hat{y}}_{i}\right)}^{2}}$$

(2) $$\mathrm{MAE}=\frac{1}{n}\sum_{i=1}^{n}\left(y_{i}-{\hat{y}}_{i}\right)$$

(3) $$R^{2}=1-\frac{\sum_{I=1}^{n}{\left(y_{i}-{\hat{y}}_{i}\right)}^{2}}{\sum_{I=1}^{n}{\left(y_{i}-\bar{y_{i}}\right)}^{2}}$$

### 2.2. Data Gathering and Model Development

In this study, SPEs and GPEs mediated with imidazolium-based polymerized ionic liquids were gathered from the literature. For developing robust ML models, a total of 120 datapoints were collected from the literature using WebPlot-Digitizer [41,42,43,44,45,46,47,48]. The collected dataset contains SMILES, temperature, and conductivity (Table 1).

**Figure 5:**
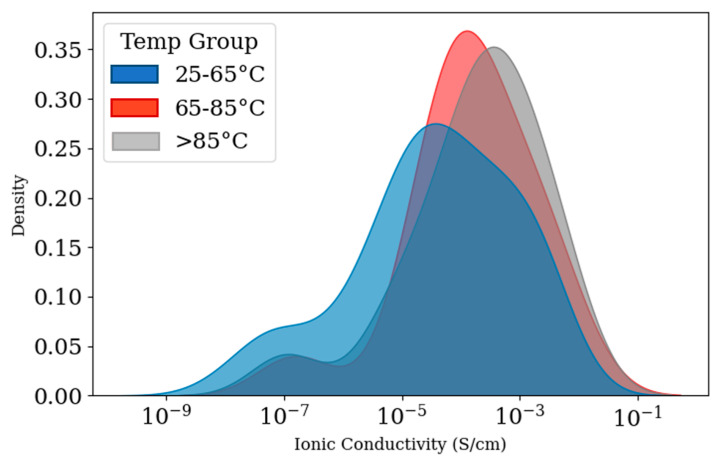
Density plot of ionic conductivity in three temperature ranges (25–65 °C, 65–85 °C, and >85 °C).

Figure 5 displays the distribution of ionic conductivity across three temperature ranges of 25–65 °C, 65–85 °C, and >85 °C. According to the density plot in Figure 5, in the temperature range of 25 to 65 °C, variability in ionic conductivity is shown, which is visible in the graph with a wide and multi-modal distribution. Also, the curve shows a long tail toward low conductivity values, with a pronounced peak around 10^−5^ S/cm. The curve for the 65–85 °C group is narrower and more concentrated than the 25–65 °C group. In this curve, fewer samples fell into the low-conductivity regime. The >85 °C group shows a very sharp and narrow peak.

By using the RDKit library [49], the molecular descriptors were generated from SMILES representations. The open-source cheminformatics toolkit RDKit allows the calculation of a wide range of chemical descriptors. Physicochemical and structural features in RDKit are calculated from SMILES strings and then used as quantitative descriptors for ML analysis. Molecular properties, including topological polar surface area, number of H_2_ bonds, and molecular weight captured by molecular descriptors, allow for a better understanding of structure-property relationships. Then ML models, including CatBoost, RF, XGBoost, and LightGBM were employed in a Python 3.11.13 environment to predict conductivity.

**Figure 6:**
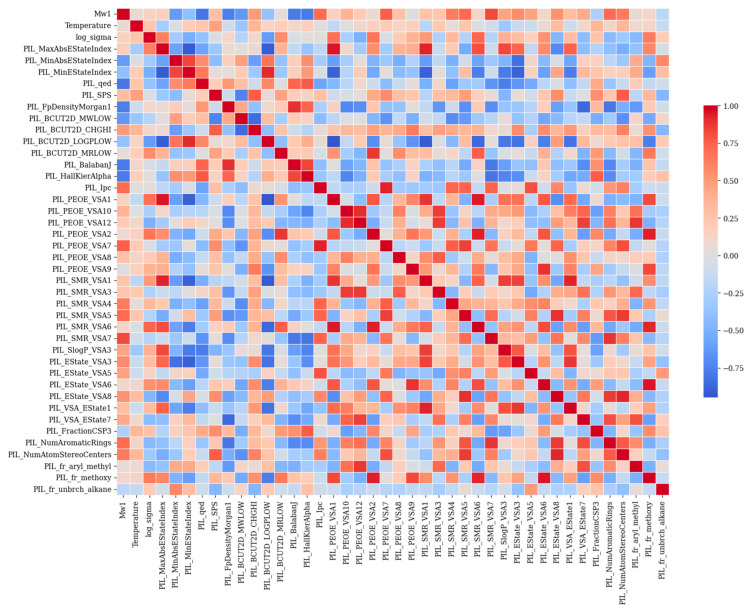
Heatmap of Pearson correlation coefficients between molecular descriptors generated by RDKit.

Based on SMILES representations, RDKit generated 434 molecular descriptors that captured electronic, structural, and physicochemical properties of the molecules. In order to eliminate the effect of highly correlated features, Pearson′s correlation coefficient was used to calculate the correlation between all pairs of descriptors. After the elimination, a total of 43 features remained. It is worth noting that the PIL prefix has been added to the descriptors. The heatmap plot in Figure 6 shows pairwise correlations between two descriptors, where each cell represents the strength of the relationship between the two features (descriptors). In this plot, each row or column depicts a pairwise linear relationship between features. In each row and column, a different feature is represented, and the colors differentiate their strength and direction based on Pearson’s correlation coefficient. When the value is close to +1, it is described in dark red, which shows a strong positive linear correlation between the two features, which means that by increasing one, the other will increase too. A value close to −1, represented in dark blue, indicates that when one feature increases, the other decreases. However, values close to 0 show little or no linear relationship. In this heatmap plot, as is evidenced, features such as PIL_PEOE_VSA, PIL, SMR_VSA, and PIL_EState have strong correlations, since they belong to similar molecular descriptor families. There are some features that have relatively medium to high correlations with the target parameter; these features are, namely, PIL_MaxAbsEStateIndex, PIL_PEOE_VSA1, PIL_PEOE_VSA2, PIL_SMR_VSA6, and PIL_fr_methoxy.

The data were standardized to make sure the data are transformed into a common scale before feeding them into ML models. On the training data, GridSearchCV and K-fold cross-validation were utilized to optimize model hyperparameters and to ensure that the random state was set for reproducible cross-validation splits. Tuning of the model hyperparameters was done by using GridSearchCV to find the best parameters for each model. Following the selection of hyperparameters, different algorithms were compared using five-fold cross-validation. The data was divided into train and test splits, 80% for train and 20% for test. Each test set included polymers that were not present in the train set. Four independent models were trained by using different random initial values, on 80% of the data. The mean R^2^, the mean RMSE, and the mean MAE were all evaluated as scoring criteria. To ensure that each polymer appeared only once in a test set, this process was repeated five times.

## 3. Results

**Figure 7:**
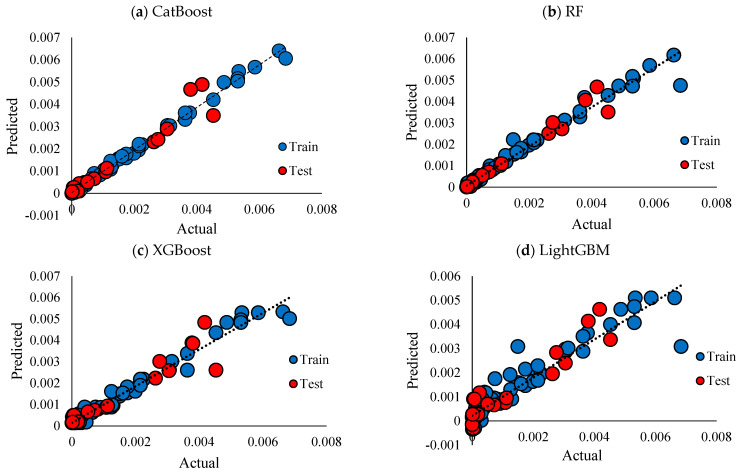
Cross plots of actual vs. predicted ionic conductivity with respect to training and testing subsets, (**a)** CatBoost, (**b**) RF, (**c**) LightGBM, (**d**) XGBoost.

**Figure 8:**
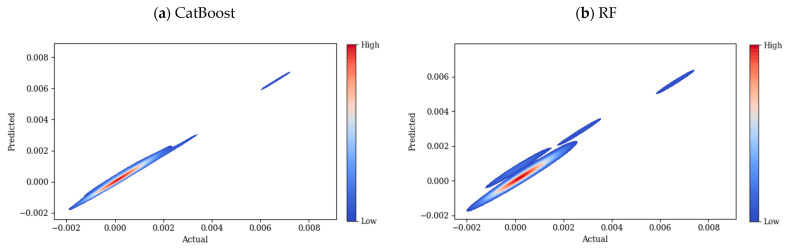

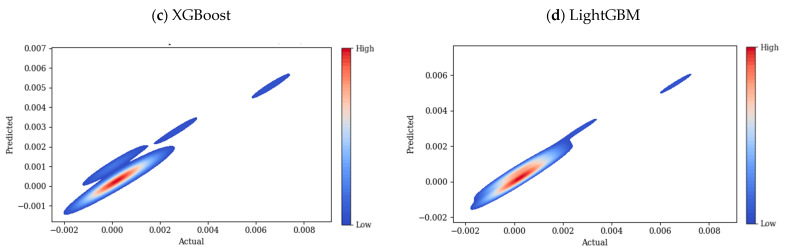
Heatmaps of prediction error for test data across four ML models: (**a**) CatBoost, (**b**) RF, (**c**) XGBoost, and (**d**) LightGBM.

R^2^, RMSE, and MAE for the employed models are presented in Table 2. CatBoost performed better than other models, followed closely by RF. The performance of XGBoost is slightly worse than that of RF, while LightGBM has the worst performance. Figure 7 compares the scatter plots of the actual versus predicted conductivity values for different employed ML models: CatBoost, RF, XGBoost, and LightGBM. In this plot, the dashed black line corresponds to the ideal prediction scenario where predicted values perfectly match the actual values (y = x). Hence, the closer the points are to the y = x line, the better the models are. For the CatBoost model Figure 7a, training data points are closely aligned with the diagonal line, testing data points also align with the diagonal line, but they are slightly more spread than the training data, which indicates some error prediction for testing data. Therefore, CatBoost performs well, showing high accuracy for both training and testing datapoints; however, the slight spread of testing datapoints suggests reliable generalization with small overfitting. In Figure 7b, the blue (training) points are aligned with the unity (y = x) line, suggesting a good fit of the model over the blue datapoints. The performance of the model over test datapoints is more scattered compared to CatBoost, which indicates higher prediction errors over unseen data. According to Figure 7c, the training datapoints no longer align well with the y = x line; the deviation from this line is further evidence for the XGBoost model (compared to the two previous models), which suggests not very good performance of this model over the training data. For testing data, this dispersion is even more pronounced, especially for higher values of conductivity, revealing that predictions might be less accurate for higher values of conductivity. Among all of the models, LightGBM performs as the least accurate model, Figure 7d, with higher deviations for training and testing datapoints. This could be a sign of the model’s difficulty in capturing complex relationships for both training and testing data points. The heatmaps in Figure 8 show the prediction errors of the test data across the four ML models: CatBoost, RF, XGBoost, and LightGBM. This plot highlights the area where the models show systematic errors. As can be seen in Figure 8a, the heatmap is mainly concentrated along the diagonal line, which is interpreted as predicted values are close to actual values. 

This plot shows a narrow spread, meaning that the model is making relatively few incorrect predictions, with only a few outliers. There are some minor deviations for higher conductivity values, but in general, the overall distribution is narrow. The systematic bias of CatBoost is minimal across different conductivity ranges. The heatmap of RF, shown in Figure 8b, is more spread, especially at medium to high conductivity values, compared to the CatBoost model. According to this plot, the model tends to underpredict high conductivity values. Similar to CatBoost and RF, the heatmap of XGBoost in Figure 8c shows a high density along the diagonal, however a few smaller clusters appear farther from the diagonal line, this could be due to subsets of data where the model performs poorly in predicting the values correctly, also the error region is wider than previous models, which shows deviations for higher conductivity values. The heatmap plot of the LightGBM model in Figure 8d shows good predictive performance, but it is more spread at higher conductivity values. Possibly due to underfitting, LightGBM and XGBoost make larger errors in higher conductivity regions. Compared to CatBoost and RF, LightGBM and XGBoost seem to struggle more with high-range values.

**Figure 9:**
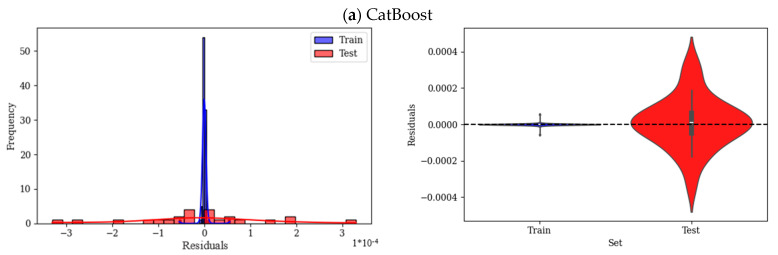

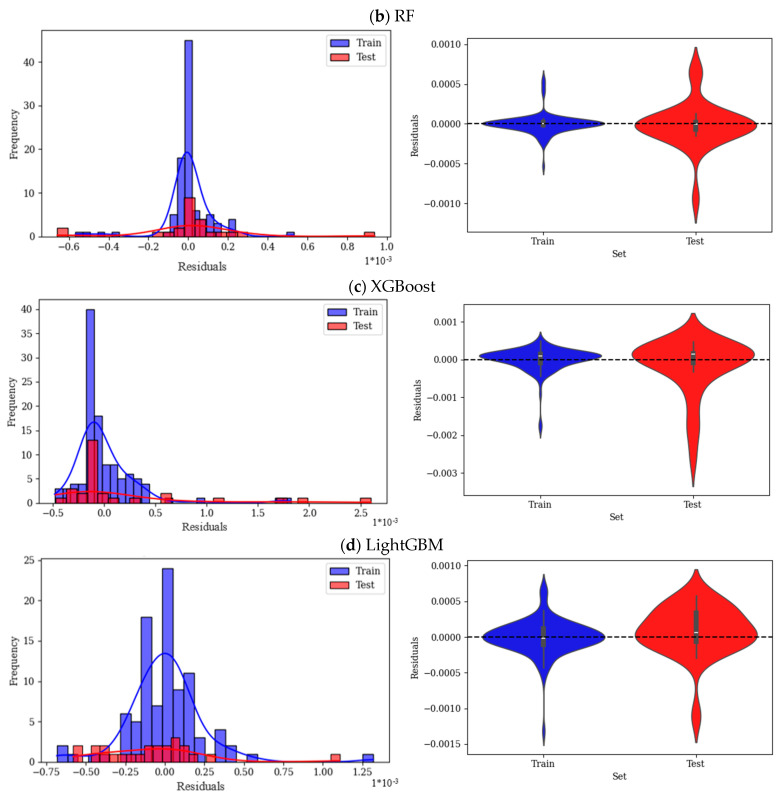
Residual analysis of ML models (**a**) CatBoost, (**b**) RF, (**c**) XGBoost, and (**d**) LightGBM.

The residual histograms and violin plots in Figure 9 provide analysis of the errors for each of the employed ML models. In residual plots, the centered and symmetric distribution indicates good performance of the models with no overprediction or underprediction. A narrower spread, skewness, or long tails show higher accuracy and systematic bias, respectively. The residual histogram plot of the CatBoost model is narrow and strongly centered around zero, as shown in Figure 9a, which highlights the small errors of this model; also, the spread of this model is symmetric, with very few residuals. The violin plot of the train residual is highly concentrated and packed around zero; however, the residual of the test data shows higher dispersion. Moreover, train residuals are tight, which shows a good fit of the model over the training data. Overall, this model exhibits minimal overfitting and good generalization with slightly higher error in the test data. In Figure 9b, the residual plot of RF shows wider spread as well as longer tails on both sides. Additionally, this model shows more residuals far from zero, which is interpreted as higher variance in errors. Violin plots of this model show a wider spread for test residuals than train residuals. In general, train residuals are highly packed, and test datapoints show higher variance. The residual histogram of XGBoost, shown in Figure 9c, has an overall wider spread than previous models; there are some clear deviations in small residuals. Moreover, the plot is shifted towards a small residual (right-skewed). The residual spread of this model, as can be seen in violin plots, is wider than that of CatBoost and RF for both train and test sets. LightGBM residuals, as shown in Figure 9d, have a normal distribution and are centered around zero, while the train data are more dispersed than the test data. Violin plot of train and test is also similar to CatBoost and RF, while it is more dispersed than those models.

### 3.1. Feature Importance

**Figure 10:**
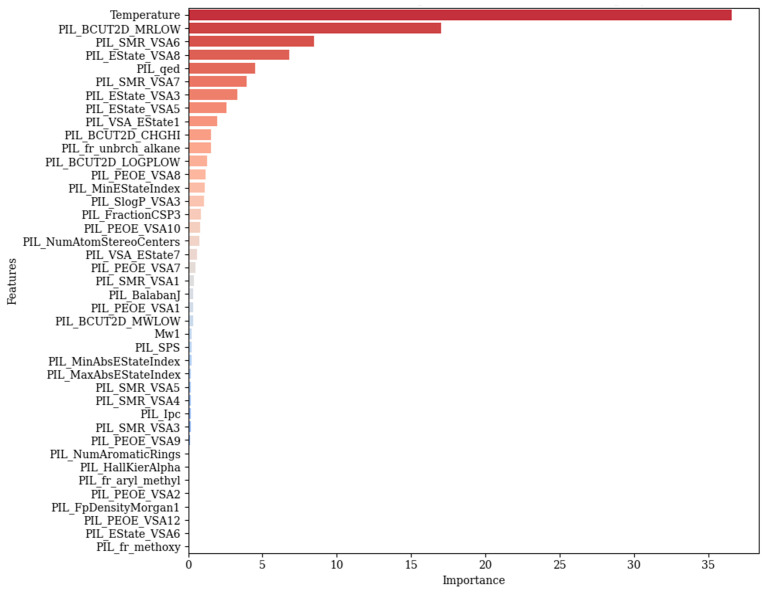
Feature importance plot for predicting conductivity employing the CatBoost model.

In this section, the analysis of the features affecting conductivity prediction was examined. For this purpose, the CatBoost model was used, which was introduced as the best model. According to Figure 10, the feature importance plot, temperature is identified as the most important variable in the prediction of conductivity. This finding is in line with physical expectations; typically, increasing temperature increases ionic mobility, which leads to higher conductivity, especially in electrolytes and polymer systems. Among the derived molecular descriptors obtained by the RDKit library, PIL_BCUT2D_MRLOW showed the second most influential feature. This parameter demonstrates a topological descriptor that is mainly based on molecular refractivity and atomic partial charges. The next important feature is PIL_SMR_VSA6, which is a descriptor that shows an approximate value of the Van der Waals surface area of atoms that have specific SMR (molar refractivity) values. In general, these two highly ranked features reflect electron distribution characteristics of molecules, size, and shape. Another important feature in conductivity prediction is PIL_EState_VSA8, which includes electrotopological state indices with the contributions of surface area. PIL_qed is the other influential descriptor, where this feature is based on properties like polarity and molecular weight. PIL_fr_unbrch_alkane, which is a measure of the unbranched alkane fragments in a molecule, highlights the importance of branching of molecules in predicting the target variable. Electronic properties, which are derived from the charge distribution, are represented by PIL_BCUT2D_CHGHI. This feature is followed by PIL_PEOE_VSA8, which contains the surface area corresponding to partial atomic charges, calculated via PEOE (partial equalization of orbital electronegativities). As it is evident from the graph, there are also some lower rank descriptors such as PIL_MinAbsEStateIndex, PIL_SlogP_VSA3, and PIL_FractionCSP3. The mentioned features show a small impact on the model’s performance, since they reflect small details about the topology of the molecules and their electronic structure. Descriptors with the least influence are placed at the bottom of the plot, which show almost zero or no importance.

### 3.2. Predicting Conductivity for Ionenes

**Figure 11:**
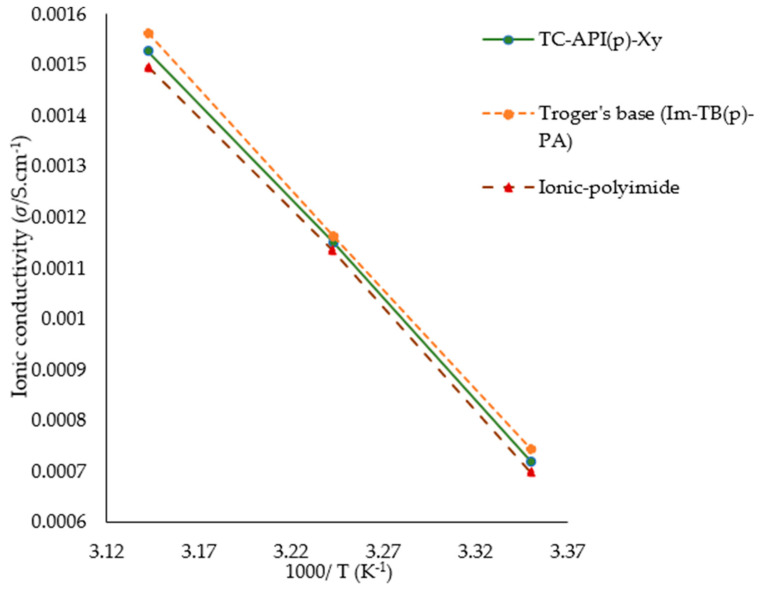
Predicted conductivity values for three ionenes using the best-performing model (CatBoost).

Figure 11 shows the predicted conductivity values of three ionenes, namely: TC-API(p)-Xy, Troger’s base (Im-TB(p)-PA), and ionic-polyimide, at random temperatures: 298, 308, and 318. This plot shows how increasing temperature can increase ionic conductivity. As it is evident in this plot, each ionene linear line is decreasing by increasing 1000/T, this can be interpreted as conductivity rises with temperature increases, which indicates that the model has learned the underlying thermally activated transport behavior. The ionic conduction in these materials is governed by Arrhenius kinetics, which implies that ion mobility increases with thermal energy. It is apparent that all the selected ionenes exhibited outstanding electrical properties with significant ionic conductivity (approaching 10^−3^ S cm^−1^). Across the entire temperature range, Troger’s base (Im-TB(p)-PA) shows the highest conductivity values. Following this, ionene TC-API(p)-Xy and ionic polyimide exhibit relatively lower conductivity values. These nearly parallel lines suggest similar activation energies for ion transport in all three materials. Vertical separation between these three lines indicates that a difference in their intrinsic conductance exists due to factors such as structural features, ion mobility, and carrier concentration. According to these results, Troger’s base may offer the best performance for applications such as electrolytes and ion-exchange membranes, which require high ionic conductivity, thereby paving the way for a new strategy in designing imidazolium ionenes with rigid backbones. In addition to capturing the overall temperature dependence of these ionenes, the ML models also captured their relative ranking, likely based on learned structural and electronic descriptors. In this regard, the model can help predict conductivity trends for new candidates of ionenes.

## 4. Conclusions

In this study, four ML models were developed for the prediction of ionic conductivity. The models were trained and tested on the data gathered from the literature. The developed models were CatBoost, RF, XGBoost, and LightGBM. A significant difference in the performance of the models for predicting conductivity values was observed. Across both training and testing datasets, CatBoost proved to be the most accurate and reliable model. Scatter plots, heatmaps, and residual plots demonstrated the superior performance of this model. According to the mentioned plots, the CatBoost model showed consistent predictions with the actual values. Following closely, RF showed excellent performance for unseen data, although it exhibited higher prediction errors at medium and higher conductivity levels. LightGBM and XGBoost performed poorly, with LightGBM having difficulty in capturing temperature-dependent trends. Ultimately, the best model (CatBoost) was used to analyze the effect of input parameters on conductivity prediction. Based on the feature importance diagram, temperature was identified as the feature with the greatest impact on conductivity. Molecular descriptors extracted from SMILES were ranked in the following order of importance, with top contributors including PIL_BCUT2D_MRLOW, PIL_SMR_VSA6, and PIL_EState_VSA8. Moreover, the best-performing model (CatBoost) was used to predict conductivity for three ionenes. Interestingly, all the selected ionenes exhibited outstanding electrical properties, with significant ionic conductivity (approaching 10^−3^ S cm^−1^). Notably, ionene with a rigid and contorted structure of Troger’s base (Im-TB(p)-PA) exhibited the highest conductivity. Additionally, the model captured the overall temperature dependency of the employed ionenes. In general, CatBoost stands out as the most robust and reliable model for conductivity prediction, making it well-suited for ionic-mediated polymers with similar applications in the future.

## Figures and Tables

**Table 1:** Collected data from the literature for conductivity prediction.

Author	PIL-Name	Number of Data	Temperature (K)	Conductivity
[41]	P(EtVIm-TFSI) (NR)	21	298.2–353.3	6.4 × 10^−6^–4.4 × 10^−4^
[42]	VEIm-TFSI	40	303.1–373.2	9.83 × 10^−9^–2.41 × 10^−4^
[43]	P−20	7	297.8–352.7	2.9 × 10^−4^–1.2 × 10^−3^
[44]	PIL-QSE	7	285.1–358.2	7.1 × 10^−4^–3.7 × 10^−3^
[45]	MIm-TFSI/EMIm-TFSI	9	301.5–363.2	1.4 × 10^−5^–6.8 × 10^−3^
[46]	PVIMTFSI-co-PPEGMA	6	333–357.8	3.8 × 10^−3^–6.6 × 10^−3^
[47]	HPILSE	23	252.9–353.2	4 × 10^−5^–5.3 × 10^−3^
[48]	PIL-GPE	7	298.1–353.2	1.2 × 10^−3^–5.3 × 10^−3^

**Table 2:** Models’ performance metrics.

	CatBoost			RF			XGBoost			LighGBM		
	Train	Test	All	Train	Test	All	Train	Test	All	Train	Test	All
R^2^	0.994	0.949	0.986	0.976	0.97	0.975	0.962	0.905	0.952	0.878	0.911	0.884
RMSE	1.2 × 10^−4^	3.35 × 10^−4^	1.87 × 10^−4^	2.55 × 10^−4^	2.57 × 10^−4^	2.56 × 10^−4^	3.2 × 10^−4^	4.5 × 10^−4^	3.55 × 10^−4^	5.81 × 10^−4^	4.41 × 10^−4^	5.56 × 10^−4^
MAE	7.33 × 10^−5^	1.83 × 10^−4^	9.52 × 10^−5^	9.5 × 10^−5^	1.26 × 10^−4^	1 × 10^−4^	2 × 10^−4^	2.54 × 10^−4^	2.14 × 10^−4^	3.54 × 10^−4^	3.28 × 10^−4^	3.4 × 10^−4^

## Data Availability

The original contributions presented in this study are included in the article. Further inquiries can be directed to the corresponding author.
